# Checkpoint and recombination pathways independently suppress rates of spontaneous homology-directed chromosomal translocations in budding yeast

**DOI:** 10.3389/fgene.2025.1479307

**Published:** 2025-04-04

**Authors:** Li Zeng, Mingzeng Sun, Michael Fasullo

**Affiliations:** ^1^ New York State Department of Public Health, Albany, NY, United States; ^2^ Ordway Research Institute, Albany, NY, United States; ^3^ School of Public of Health, University at Albany, Albany, NY, United States

**Keywords:** chromosomal translocations, homologous recombination, cell cycle checkpoints, radiation, budding yeast

## Abstract

Homologous recombination between short repeated sequences, such as Alu sequences, can generate pathogenic chromosomal rearrangements. We used budding yeast to measure homologous recombination between short repeated *his3* sequences located on non-homologous chromosomes to identify pathways that suppress spontaneous and radiation-associated translocations. Previous published data demonstrated that genes that participate in *RAD9*-mediated G_2_ arrest, the S phase checkpoint, and recombinational repair of double-strand breaks (DSBs) suppressed ectopic recombination between small repeats. We determined whether these pathways are independent in suppressing recombination by measuring frequencies of spontaneous recombination in single and double mutants. In the wild-type diploid, the rate of spontaneous recombination was (3 ± 1.2) × 10^−8^. This rate was increased 10–30-fold in the *rad51*, *rad55*, *rad57, mre11, rad50, and xrs2* mutants, seven-fold in the *rad9* checkpoint mutant, and 23-fold in the *mec1-21* S phase checkpoint mutant. Double mutants defective in both *RAD9* and in either *RAD51*, *RAD55*, or *RAD57* increased spontaneous recombination rates by ∼40 fold, while double mutants defective in both the *MEC1* (ATR/ATM ortholog) and *RAD51* genes increased rates ∼100 fold. Compared to frequencies of radiation-associated translocations in wild type, radiation-associated frequencies increased in *mre11*, *rad50*, *xrs2*, *rad51*, *rad55* and *rad9 rad51* diploid mutants; an increase in radiation-associated frequencies was detected in the *rad9 rad51* diploid after exposure to 100 rads X rays. These data indicate that the S phase and G_2_ checkpoint pathways are independent from the recombinational repair pathway in suppressing homology-directed translocations in yeast.

## Introduction

Homologous recombination between short repeated sequences can generate pathogenic chromosomal rearrangements; such recombination is often referred to as ectopic recombination. In mammalian cells, recombination between Alu sequences generates intrachromosomal deletions and translocations; several such rearrangements are associated with leukemias (Jeffs, et al., 1998; Strout et al., 1998; Balachandran et al., 2022). In budding yeast, recombination between delta sequences generates intrachromosomal deletions and inversions (Rothstein et al., 1987). While such events can spontaneously occur, they can also be stimulated by radiation and genotoxic agents, which lead to either replication fork collapse or double-strand breaks (DSBs, for review, see Nickoloff et al., 2023).

Eukaryotic cells have evolved genetic pathways to suppress ectopic recombination between repeats to minimize the occurrence of pathogenic rearrangements after exposure to environmental insults of DNA replication stress. These pathways include those that directly participate DNA repair and those that participate in cell-cycle checkpoint pathways which delay or arrest the cell cycle so that repair of DNA damage occurs before cellular division (Hartwell et al., 1994). Together these pathways assure that sister chromatids are the preferred substrates for recombinational repair of DSBs (Kadyk and Hartwell, 1992) and thus minimize ectopic recombination events that could generate rearrangements.

In budding yeast, DNA repair genes that function in DSB-repair by homologous recombination proteins constitute the *RAD50* epistasis group (for review, see Krogh and Symington, 2004). Encoded proteins function to resect the ends of DSBs to generate recombinogenic 3′ single-strands, which in turn, are substrates for Rad51 filament formation, which catalyzes DNA strand invasion and the formation of Holliday intermediates. Additional proteins catalyze chromatin remodeling and resolve recombination intermediates. A recurrent theme is the presence of multiple Rad51 paralogs, nucleases, and resolvases. The identification of multiple mammalian orthologs and paralogs corresponding to yeast genes underscore the conservation of recombination functions in eukaryotic organism (Liu et al., 1998). Furthermore, defects in multiple mammalian recombination genes, such as BRCA1 and BRCA2, have been linked to cancer, underscoring the importance of elucidating the role of multiple recombination genes in maintaining genetic stability (for review, see Rein and Bernstein, 2023).

Checkpoint pathways have been divided into those that function at distinct stages in the cell cycle including the G_1_-S, S phase, and G_2_ phase. In budding yeast, the essential yeast gene, *MEC1* (Kato and Ogawa, 1994), is the ataxia telangiectasia mutated and *RAD3* related (ATR) ortholog, whose activation is facilitated by adaptors *RAD9* and *MRC1* (Sanchez et al., 1996). Downstream protein kinases are then required for the transcriptional DNA damage response, S phase delay, and the arrest of the cell cycle at the G_2_/M transition. *MEC1*’s signaling function in facilitating homologous recombination (Sanford et al., 2021; Xie et al., 2024) involves phosphorylation of key proteins, including Sgs1 (Hegnauer, et al., 2012), Rad51 (Flott et al., 2011) and Rad55 (Herzberg et al., 2006).

The role of genes in both the *RAD50* epistasis group and checkpoint pathways in suppressing gross chromosomal rearrangements (GCRs) and intrachromosomal rearrangements has been well-documented. Importantly, knocking down both *RAD51* and *MEC1* confers a synergistic increase in GCRs (Pennaneach and Kolodner, 2004; Putnam and Kolodner, 2017), and DSBs are potent stimulators of GCRs (Myung and Kolodner, 2003). These studies underscore the importance of the S phase checkpoint in suppressing GCRs. However, these studies suggest that the checkpoint gene, *RAD9*, plays a minor role in suppressing GCRs (Myung et al., 2001).

Fasullo et al. (1998) illustrated the role of *RAD9* in suppressing both spontaneous and DNA damage-associated homology-directed chromosomal translocations. These studies were performed in diploid and not haploid strains. The role of cell cycle arrest in suppressing radiation-associated chromosomal rearrangements was evident by observations that arresting *rad9* mutants with the microtubule inhibitor nocodazole decreased elevated frequencies of radiation-associated translocations. Additional studies have shown the role of both *RAD51* (Fasullo et al., 2004) and *MEC1* (Fasullo et al., 2010) in suppressing homology-directed spontaneous and DNA damage-associated rearrangements. In these studies, electrophoretic karyotypes of recombinants revealed half-reciprocal translocations and additional rearrangements, suggesting that multiple recombination events occurred in mutant strains.

In this study, frequencies of homology-directed chromosomal rearrangements were measured in both single and double mutants defective in *RAD9*-mediated checkpoint function, S phase checkpoint function and recombinational repair. The overarching conclusion is that double mutants defective in both the recombinational DNA repair genes and either *RAD9*-mediated or S phase checkpoint function exhibit synergistic increases in rates of spontaneous recombination. Further experiments were performed to determine whether *rad* and checkpoint single and double mutants exhibit enhance radiation-associated recombination. We suggest that double mutants may be optimal strains for identifying effects of low dose radiation and the genotoxicity of environmental agents.

## Methods

### Growth media and transformations and yeast strains

Standard media were used for the culture of yeast and bacterial strains (Sherman et al., 1986). LB-AMP (Luria broth containing 100 μg/mL ampicillin) was used for the culture of the *Escherichia coli* DH1 strains. Media used for the culture of yeast cells included YPD (yeast extract, peptone, dextrose), SC (synthetic complete, 2% dextrose), SC-HIS (SC lacking histidine), and SC-ADE (SC-lacking adenine). YPD-Kan (G418) plates contain YPD supplemented with 50 μg/mL G418 (Sigma). YP(A)D contains YPD with 80 mg/L adenine. Gene knockouts were performed by one-step gene replacement (Rothstein, 1983), using standard lithium acetate transformation protocols (Gietz et al., 1995).

All the yeast strains, including BY4741 (Brachmann et al., 1998), and YA148 (Lesser and Guthrie, 1994) are of the S288c genetic background. The wild-type diploid strain containing the *his3* recombination substrates (Fasullo and Davis, 1987) on one copy of chromosomes II and IV has been previously described. Checkpoint and recombination mutants were constructed by gene disruptions and genetic crosses. Essentially, two sets of isogenic haploid strains were constructed; one set contains the recombination substrates, and the other does not. The homozygous diploid strains, *rad9* (YB134), *rad51* (YB170), and *mec1-21* (YB325), which also contain the recombination substrates, have been previously described (Fasullo et al., 1998; Fasullo et al., 2001; Fasullo et al., 2010). Additional homozygous diploid strains were obtained by knocking out *RAD51*, *RAD55 RAD57*, *RAD50*, *RAD54*, *MRE11*, *XRS2* in respective *MAT* **a** and *MAT*α haploid strains, using plasmids pRAD51Δ (Shinohara et al., 1992), pST11 (Lovett and Mortiner, 1987), pSM51 (Schild), pNKY83 (Alani et al., 1989), pSM31 (Schild), pSK-MRE11delta (Bressan et al., 1999), pET139 (Ivanov et al., 1994), respectively. The *rad9 rad51*, *rad9 rad50*, *rad9 mre11*, *rad9 rad54* double mutants were constructed by knocking out the *RAD* genes in *MAT*a *rad9* and *MAT*α *rad9* haploids. The *rad51 rad1* double mutants were obtained by knocking our *RAD51* in *rad1* haploid strains, and the diploid was obtained by mating the haploid double mutants. The *rad51 rad9* double mutants were made by genetic crosses and mating the haploid double mutants. All disruptions were confirmed by radiation sensitivities and the haploid strains were then mated to generate the diploid strains used in this study. The radiation sensitivities of diploid mutants were confirmed by exposing inoculated YPD plates to 60 J/m^2^ and 6 krads of X rays and imaging after 3 days of 30°C incubation (see Supplementary Figure S1). PCR was used to confirm *rad59*::KanMX gene disruptions. The presence of the *his3-Δ200* allele was confirmed by PCR analysis using the primers 5′-CAC​GGC​AGA​GAC​CAA​TCA​GTA-3′ and 5′-GCA​CTC​CTG​ATT​CCG​CTA​ATA-3’.

Haploids containing the recombination substrates for measuring unequal sister chromatid recombination (uSCR) and diploid strains containing *ade2-a* and *ade2-n* for measuring homolog recombination were previously described (Fasullo et al., 2010). The *mec1-21 rad51* haploid strain containing the *his3* recombination substrates was constructed by knocking out *RAD51* in the *mec1-21* haploids. Construction of the *mec1-21* mutant diploid strains has been previously described (Fasullo et al., 2010). The *mec1-21 rad9* double mutant was constructed by knocking out *RAD9* in the *MAT* **a** and *MAT*α *mec1-21* haploids and then mating the haploid double mutants.

### Determining the rates of spontaneous recombination

The rates of spontaneous, mitotic events that generate either SCE, heteroallelic or translocations were determined by the method of the median (Lea and Coulson, 1949), as performed by Esposito et al. (1982), using 11 independent colonies for each rate calculation. This method is essentially the same as that employed by Spell and Jinks-Roberston (2004). In all rate calculations, the number of cell divisions was estimated by the number of viable cells that formed colonies. Statistical analysis difference was determined by the by the Mann-Whitney *U* test, where there are at least two independent calculations for each mutant strain (Zar, 1996). To measure the rates of homolog recombination between *ade2* heteroalleles, colonies were obtained from cells inoculated on YP(A)D solid medium to suppress expression of the adenine pathway. To determine whether the rate of recombination increased in the diploid mutants, compared to the single mutants, the interaction factor was calculated according to David et al. (2016), see Supplementary Table 3.

### UV and X ray stimulation of recombination

The number of His^+^ recombinants stimulated by DNA-damaging agents was determined by subtracting the spontaneous frequency from the stimulated frequency and multiplying by 10^7^, the approximate number of cells plated, as done previously (Fasullo et al., 1994). The significance of the differences between mutants and Rad^+^ (diploid) strains was determined by using the two-tailed paired sample *t*-test (Zar, 1996). Protocols used to assess the recombinogenicity of UV and X rays have been described elsewhere (Fasullo et al., 1994). The X-ray radiation source was purchased from Rad Source, Inc. (Wheeling, Ill.), and the dose rate was 440 rads/min. For measuring radiation-associated stimulation of translocations, cells were washed twice in sterile H_2_O, resuspended in H_2_O, irradiated, and then plated on selective medium (SC-His) for selecting for recombinants and inoculating an aliquot of the appropriate dilution on YPD medium to measure viability. Typically, 20–200 colonies were counted on SC-HIS plates, and 100–300 colonies were counted on YPD plates. The UV source emitted 260 nm UV light at a dose rate of 2 J/m^2^. One-way ANOVA followed by Dunnet’s test was used to determine the statistical significance of differences between radiation-associated frequencies (Zar, 1996).

## Results

### *RAD51*, *RAD51* paralogs or *MRX* genes suppress rates of spontaneous homology-directed translocations

**FIGURE 1:**
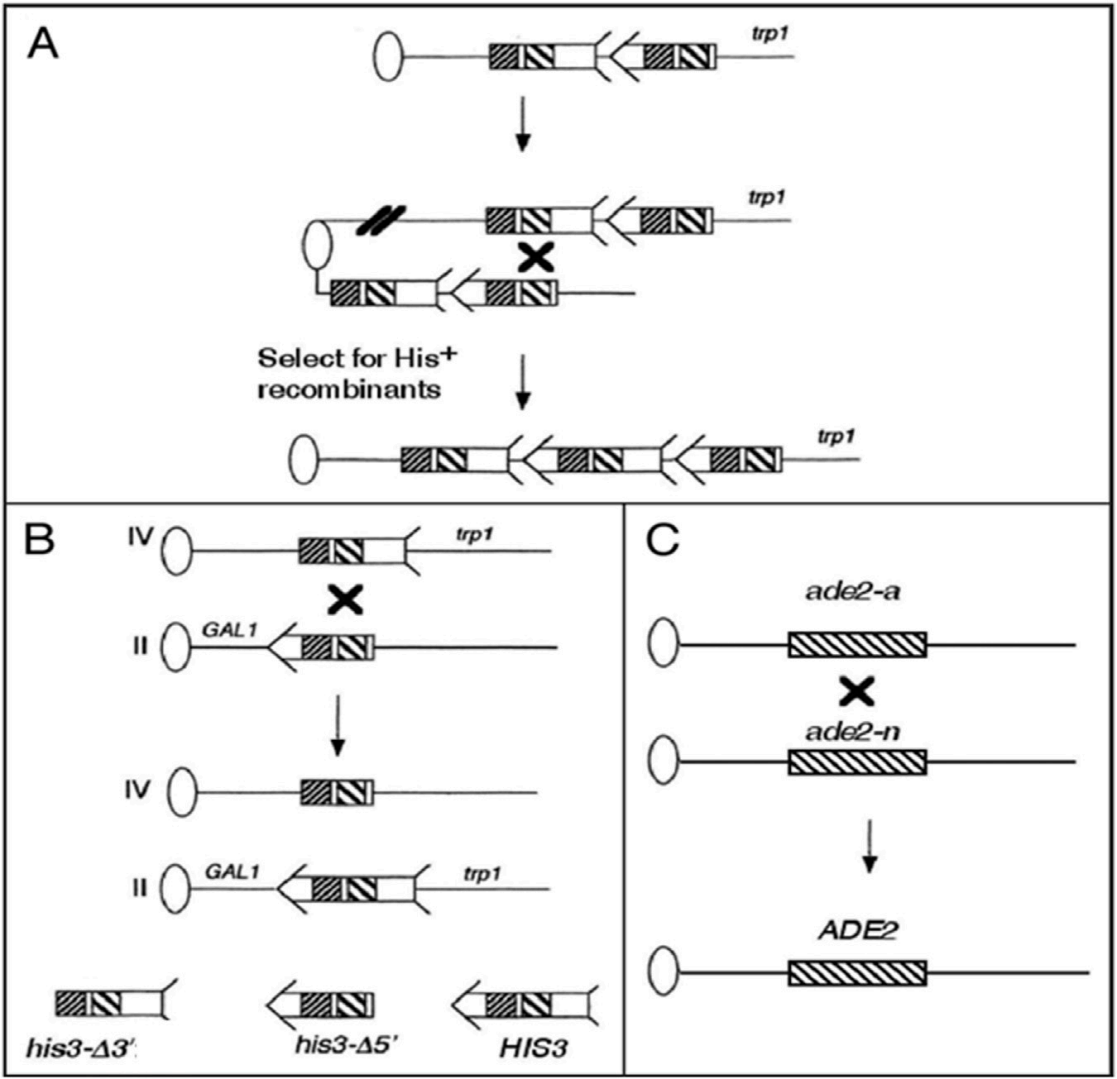
Sister chromatid, translocation, and heteroallelic recombination assays used in this study. Ovals represent centromeres and lines represent chromosomes. For simplicity, the left arms of the chromosomes are not included. An arrow and feathers together denote *HIS3*. As indicated in the bottom left of the figure, the 5′ deletion, *his3-Δ5′*, lacks the feather and the 3′ deletion, *his3-Δ3′*, lacks the arrow. The two regions of the sequence identity shared by the *his3* fragments are indicated by decorated boxes; closely spaced diagonal-filled boxes indicate a region of 167 bp, and the broadly spaced diagonal line-filled boxes indicate a region of ∼300 bp. The “X” indicates where recombination occurred. **(A)** The *his3*-truncated fragments are integrated at the *TRP1* locus to measure uSCR events. His^+^ recombinants resulting from unequal SCE were selected that contain *HIS3* flanked by *his3-Δ3′* and *his3-Δ5'*. **(B)** Homology-directed translocation events result from recombination between the same his3 fragments located each on chromosomes II and IV. Positions of the *GAL1* and *trp1* are shown on chromosomes II, IV, and the chromosomal translocations. **(C)** Homolog recombination between *ade2-a* and *ade2-n* generates *ADE2*. *ADE2* and *ade2* alleles are represented as boxes; *ade2-a* and *ade2-n* are separated by approximately 1 kb. Figure is an adaptation from Fasullo and Sun, (2008b).

Rates of spontaneous, mitotic translocations were measured in diploid and haploid strains using *his3* constructs that are shown in Figure 1. We measured rates of spontaneous translocations in both diploid single and double mutants that were defective in *RAD51*, *RAD55*, *RAD57* and the checkpoint gene *RAD9*. The rate of spontaneous translocations in the Rad^+^ diploid was 3 × 10^−8^ and was similar to previous measurements (4 × 10^−8^, average) obtained from three independent Rad^+^ diploids (Fasullo et al., 1998; Fasullo et al., 2004; Fasullo et al., 2010). Knocking out either *RAD51*, *RAD55*, or *RAD57* conferred a ten-fold or greater increase in translocation frequency (Table 1). Diploid mutants defective in *MRE11*, *RAD50,*or *XRS2*, which encode the MRX complex, exhibited a 22–27-fold increase in the rate of homology-directed translocations. However, knocking out *RAD54*, only conferred a two-fold increase in recombination, compared to wild type, which was not significant (P > 0.05, Mann-Whitney), while knocking out *RAD59*, conferred a two-fold decrease; *RAD54* functions at multiple stages in the initiation and processing of recombination intermediates (Heyer et al., 2006). These data indicate that knocking out the *RAD51* paralogs, *RAD55* and *RAD57*, confers a similar phenotype as that of knocking out *RAD51,* and that knocking out genes encoding the MRX complex conferred the highest increase in translocation frequencies among the *RAD50* group. Thus, specific genes that function in recombinational repair of DSBs suppress homologous recombination between short repeated sequences on non-homologous chromosomes.

**TABLE 1:** Rates of spontaneous translocations in diploid mutants defective in either the *RAD9* checkpoint or in recombinational repair.

Relevant genotype^a^	Number of experiments	Rate of translocations x 10^−8^ ^b^	Ratio^c^
Wild type (YB110)	5	3 ± 1.2	1
Double-strand break repair			
*rad51* (YB170)	3	34 ± 9	11
*rad55* (YB744)	3	37 ± 6	12
*rad57* (YB745)	3	38 ± 8	13
*rad54* (YB741)	2	6.8 ± 0.1	2
*rad50* (YB746)	3	67 ± 10	22
*mre11* (YB743)	2	85 ± 7	28
*xrs2* (YB747)	2	80 ± 14	27
*rad59*(YB748)	4	1.5 ± 0.4	0.5
*rad51 rad59* (YB752)	2	7 ± 5	2
*rad9* and double mutants			
*rad9* (YB134)	5	21 ± 5	7
*rad9 rad51* (YB749)	2	180 ± 23	57
*rad9 rad55* (YB753)	3	230 ± 62	77
*rad9 rad57* (YB754)	3	203 ± 30	68
*rad9 rad54* (YB751)	2	165 ± 50	55
*rad9 xrs2* (YB755)	2	130 ± 14	43
*rad9 mre11* (YB756)	2	170 ± 57	57
*rad9 rad50* (YB750)	2	58 ± 11	19
*rad9 rad59* (YB762)	2	10 ± 0	3

^a^ All strains (strain numbers) are diploid strains; for full genotype see Supplementary Table S1.

^b^ Number of events per cell division, as measured by the method of the median.

^c^ Ratio = rate of recombination in mutant/rate of recombination in wild type; numbers are rounded.

### Rates of homology-directed translocations are synergistically increased in mutants defective in both *RAD9* and recombinational repair

Checkpoint and DNA repair mutants exhibit higher rates of spontaneous translocation events in diploid cells; however, it is unclear whether these pathways function independently to suppress recombination. We therefore determined whether knocking out both checkpoint and recombination genes would lead to a synergistic increase in recombination. Failure to arrest the cell cycle in G_2_ can also increase the frequency of DNA damage-associated chromosomal rearrangements. Compared to wild type, the *rad9* diploid mutants exhibit a seven-fold increase in the rate of spontaneous recombination, similar to previous reports (Fasullo et al., 1998). We measured rates of translocations in double mutants defective in both *rad9* and in recombinational repair genes (Table 1). The rate of translocations in *rad9* mutants defective in either *RAD51*, *RAD55*, or *RAD57* were similar and 57–77-fold higher than the wild-type rate. We also observed a 55-fold increase in rate of translocations in *rad9 rad54* double mutant, compared to the rate in wild type. The fold increase in rates of the double mutant in comparison to the single mutants was thus synergistic, *i.e.*, the increase was greater than additive (Perez-Perez et al., 2009), as indicated by the positive IF (see Supplementary Table 3). However, the synergistic increase in recombination is dependent on ploidy; the rate of spontaneous translocations was (2.0 ± 0.2) × 10^−8^ in the *rad9 rad51* haploid, a rate below 5.4 × 10^−8^, observed in *rad9* haploids. The higher rate of spontaneous recombination observed in *rad51* diploids is also dependent on *RAD59*, indicating that *RAD59* has a role in *RAD51*-independent ectopic recombination between these short-repeated sequences. This indicates that recombinational repair and *RAD9* checkpoint and *RAD51* participates in independent pathways for suppressing homology-directed translocation events in diploid strains.

The MRX complex is required in checkpoint signaling (Grenon et al., 2001). We also measured the rates of spontaneous recombination in *mre11*, *xrs2*, and *rad50* diploid mutants that were defective in the *RAD9* checkpoint function (Table 1). While *rad9 mre11* and *rad9 xrs2* double mutants exhibited over a 40-fold increase in rates of translocations., rates of translocations in *rad50* and *rad9 rad50* were still in the range of 20–25 ‐fold increase, compared to wild type. However, the radiation sensitivities of the *rad9 mre11*, *rad9 xrs2*, and *rad9 rad50* mutants were uniformly higher than that of the *mre11*, *xrs2*, and *rad50* single mutants (Supplementary Figure S1), and all the double mutants generate microcolonies after irradiation (Supplementary Table S2). Similarly, while there is a noticeable delay in the doubling time for the *mre11*, *xrs2*, and *rad50* single mutants (100–110 min), the doubling time for the *rad9 mre11*, *rad9 xrs2*, and *rad9 rad50* mutants was approximately the same as wild type (80–90 min). The data indicate that knocking out *RAD9* in the *mre11*, *xrs2* and *rad50* single mutants increases radiation sensitivity while increasing the rate of translocations in either *mre11* or *xrs2* but not in *rad50* mutants.

### The *MEC1*-mediated S phase checkpoint and *RAD51* independently suppress homology-directed translocations

Previous studies had shown that defects in S phase checkpoint function also increase translocations that result from homologous recombination between short repeats. The *mec1-21* mutant (Craven and Petes, 2000) is a *mec1* hypomorph that is defective in S phase checkpoint function but retains some G_2_ checkpoint function (Sun and Fasullo, 2007). We had previously measured recombination in the diploid *mec1-21* mutant and observed a 23-fold increase in rates of spontaneous translocations, compared to the rate observed in the wild-type diploid (Fasullo et al., 2010). After *RAD51* was knocked out in the *mec1-21* diploid, the rate increased to 2.9 × 10^−6^, which is approximately 100-fold higher than what is observed in the wild-type diploid (Table 2). The rate of spontaneous translocations in the *mec1-21 rad51* diploid is similar to the rate of spontaneous unequal siter chromatid exchange between juxtaposed *his3* recombination substrates observed in Rad^+^ strains (Dong and Fasullo, 2003). However, knocking out *RAD9* in the *mec1-21* diploid decreased the rate of translocations, compared the rate observed in *mec1-21*, although it was still higher than wild type (Fasullo and Sun, 2008a). These data indicate that *MEC1* and *RAD51* participate in independent pathways for suppressing homology-directed chromosomal rearrangements. These data are consistent with the notion that genetic instability phenotypes are highest in S phase checkpoint mutants (Myung et al., 2001; Myung et al., 2004; Pennaneach and Kolodner, 2004).

**TABLE 2:** Rates of spontaneous translocation, heteroallelic, and sister chromatid recombination events in *rad51*, *mec1-21*, and *rad9* single and double mutants.

Genotype^a^	Translocation (×10^−8^)^b^ ^.^ (diploid Strain)	Ratio^c^	Heteroallelic (×10^−6^)^2^	Ratio^c^	Sister Chromatid (×10^−6^)^b^ (Haploid strain)	Ratio^c^
*MEC1*	3.0 ± 0.8 (YB348)	1.0	0.9 ± 0.02	1.0	1.1 ± 0.1 (YB163)	1.0
*mec1-21*	68 ± 16 (YB325)	23	9.1 ± 1.9	10	6.3 ± 0.9 (YB311)	5.7
*rad51*	33 ± 9 (YB742)	11	<0.01	0.01	2.3 ± 0.9 (YB177)	2
*mec1-21 rad51*	291 ± 16 (YB757)	100	1 ± 0.1	1.0	3.4 ± 1.9 (YB758)	3.1
*rad9*	21 ± 5 (YB134)	7	0.7 ± 0.2	0.8	1 ± 0.2 (YB147)	1.0
*mec1-21 rad9*	19 ± 5(YB756)	6	3.8 ± 1	4	1.8 ± 1(YB759)	1.0

^a^ For full genotype, see Supplementary Table S1; strains to measure frequencies of translocations and heteroallelic recombination are diploids; those that measure sister chromatid exchange are haploids. The same diploid strain was used to measure frequencies of translocations and heteroallelic recombination.

^b^ Recombination events per cell division, as measured by the method of the median (Lea and Coulson, 1949); N ≥ 2 for each strain. Rates of spontaneous of translocations for *rad9* and *mec1-21 rad9* were previously published (Fasullo and Sun, 2008b).

^c^ Ratio = rate of recombination in mutant/rate of recombination in wild type.

We previously observed that the rates of spontaneous unequal sister chromatid recombination (uSCR) and homolog recombination between *ade2* heteroallelic increased in *mec1-21* mutants, compared to wild type (Table 2; Fasullo et al., 2010). While there is no significant difference in the rates of spontaneous uSCR in *rad51* mutants and wild type, the rate of spontaneous, homolog recombination between *ade2* heteroalleles cannot be detected (<1 × 10^−8^), in agreement with results obtained by Bai and Symington (1996). However, in diploid *mec1-21 rad51* mutants recombination between *ade2* heteroalleles was detected, and in the *mec1-21 rad51* haploid the spontaneous rate of uSCR was three-fold higher than wild type. These data indicate that multiple types of homologous recombination events are enhanced in the *mec1-21* strain, which are variably affected by knocking out *RAD51*.

Knocking-out *RAD9* had no significant effect on the rates of unequal uSCR and homolog recombination between heteroalleles, consistent with observations that *rad9* mutants do not generally exhibit mitotic phenotypes (Weinert and Hartwell, 1990). Similar to the *RAD9* requirement for the high translocation frequencies observed in the *mec1-21* diploid, knocking out *RAD9* in diploid and haploid *mec1-21* mutants also decreased homolog recombination and uSCR, respectively (Table 2). These data indicate diverse types of mitotic recombination events are increased in *mec1-21* mutants which are variably affected by knocking out *RAD51* and *RAD9*.

### Both *rad51* and *rad55* diploid mutants exhibit enhanced radiation-associated recombination

**FIGURE 2:**
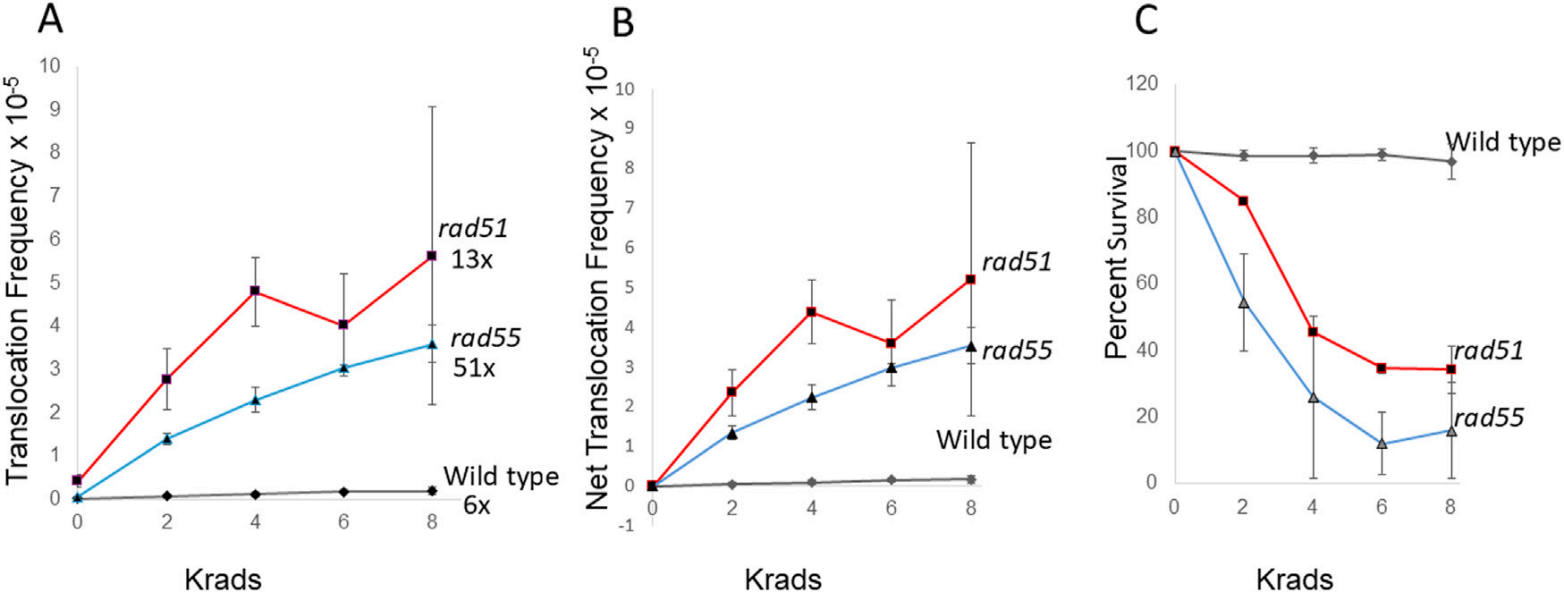
Stimulation of homology-directed translocations by ionizing radiation in wild type, *rad51* and *rad55* diploid strains. Radiation-associated recombination frequencies (translocations) were measured after *rad51* (N = 3) and *rad55* (N = 2), and wild type (N = 5) diploid strains were exposed to 0, 2, 4, 6, and 8 krads X rays. Recombination frequencies **(A)** were plotted against radiation dose. Net recombination frequencies were calculated by subtracting the spontaneous frequency from the radiation-associated frequency for each experiment **(B)**. The average survival percentage was plotted against radiation dose **(C)**. Shaded triangles represent the *rad51* strain (YB170), shaded triangles represent the *rad55* strain (YB744), and shaded diamonds represent the wild-type strain (YB110). Fold change was calculated by dividing 8 krad-associated frequency by the spontaneous frequency in **(A)**.

The DNA lesions that initiate spontaneous translocation events are unknown. One explanation for the higher relative rates of spontaneous translocations in *rad51* and *rad55* diploid mutants is that spontaneously generated DSBs stimulate more homology-directed translocations in these *rad* mutants compared to those stimulated in wild type. In addition, both *rad51* and *rad55* mutants are defective in DNA damage-associated uSCR (Dong and Fasullo, 2003). We therefore exposed *rad51* and *rad55* diploid mutants to 2, 4, 6, and 8 krads of X rays and measured frequencies of X-ray associated translocations (Figure 2). For all exposures, the X-ray-associated frequencies of translocations in the *rad* mutants were significantly higher than wild-type frequencies (P < 0.05). We observed a dose-dependent increase in the frequencies of recombination wild type, *rad51*, and *rad55* mutants (P < 0.05); however, the dose-dependent differences were most significant for the *rad55* mutant (P < 0.01, Dunnett’s). Compared to the frequencies of spontaneous recombination, translocation frequencies after 8 krad exposure were 13-fold and 51-fold higher for *rad51* and *rad55*, respectively, compared to 7-fold for wild type (Figure 2). The data indicate that frequencies of X-ray-associated translocations are similar in both the *rad55* mutants and *rad51* mutants and suggest that knocking-out other *RAD51* paralogs may also confer increased X-ray associated genetic instability.

### *mre11* diploid mutants exhibit more UV and X ray-associated recombination compared to wild type

**FIGURE 3:**
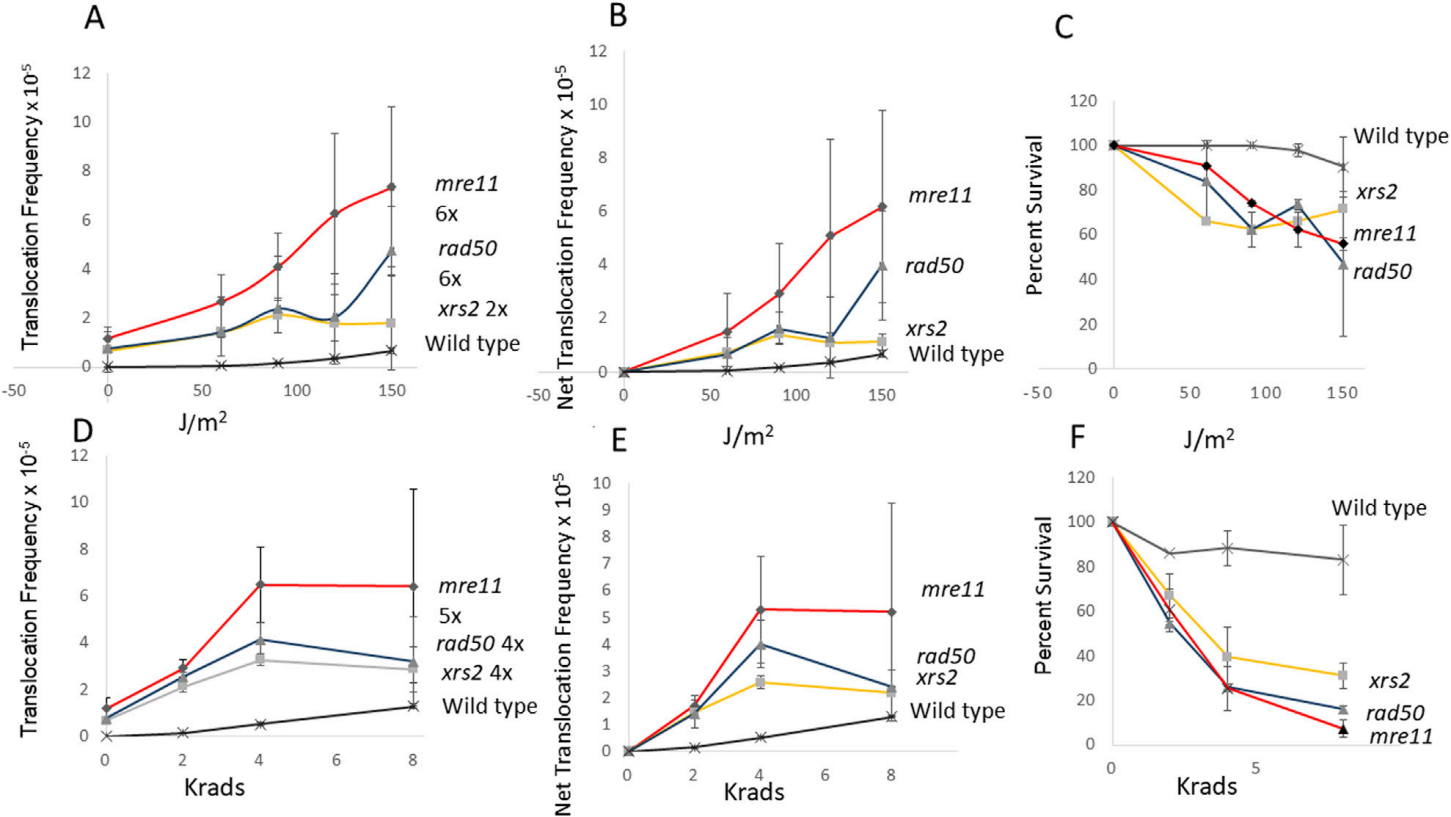
Stimulation of homology-directed translocations by UV and ionizing radiation in wild-type, *mre11*, *rad50*, and *xrs2* diploid strains. In panel **(A)**, UV-associated recombination frequencies (translocations) were measured after wild type (N = 2), *mre11* (N = 4), *rad50* (N = 2), and *xrs2* (N = 3) diploid strains were exposed to 60, 90, 120 and 150 J/m^2^ UV. Net recombination frequencies were calculated by subtracting the spontaneous frequency from the radiation-associated frequency for each experiment **(B)**. The average survival percentage was plotted against radiation dose **(C)**. X-ray associated recombination were measured after wild type (N = 2), *mre11* (N = 4), *rad50* (N = 2), and *xrs2* (N = 3) diploid strains were exposed 0, 2, 4, and 8 krads X rays (Panel D). Recombination frequencies **(D)** were plotted against radiation dose. Net recombination frequencies were calculated by subtracting the spontaneous frequency from the radiation-associated frequency for each experiment **(E)**. The average survival percentage was plotted against radiation dose **(F)**. Filled diamond represents the *mre11* strain (YB743), filled triangle represents the *rad50* strain (YB746), and the filled box represents the *xrs2* strain (YB747), and the star represents the wild-type strain. (YB348). Fold change was calculated by dividing radiation-associated frequency by the spontaneous frequency in **(A, D)**.

Considering that *mre11*, *rad50*, and *xrs2* diploid mutants also exhibit higher rates of spontaneous translocations, compared to wild-type diploid, we measured the radiation-associated translocations frequencies were higher in *mre11*, *rad50,* and *xrs2* mutants (Figure 3). We exposed wild type, and *mre11*, *rad50,* and *xrs2* mutants to both 2, 4, and 8 krads, as well as 60, 90, 120, and 150 J/m^2^. While the radiation-associated frequencies of translocations in wild type are significantly lower than all those in the *rad* mutants (P < 0.05), there is more than a 100-fold increase in UV-associated frequencies (6.8 × 10^−6^, avg.) and X-ray associated frequencies (1.3 × 10^−5^, avg.) in wild type, compared to the non-irradiated wild-type control (6 × 10^−8^, avg.). The frequencies of X-ray and UV-associated translocations were highest in the *mre11* mutants and sixfold higher than the frequencies obtained from the non-irradiated control (Figure 3); the dose dependence is significant for both X-ray and UV exposures (P < 0.05, Dunnett’s). On the other hand, the frequencies of X-ray and UV-associated translocations are similar in the *xrs2* and *rad50* mutant, and while frequencies of UV and X-ray associated frequencies was dose-dependent for the *xrs2* mutant (P < 0.05, Dunnett’s), only the X-ray associated frequencies were significant for the *rad50* mutants (P < 0.01, Dunnett’s). These data illustrate that ectopic recombination between homologous sequences can be stimulated by radiation in *mre11, rad50,* and *xrs2* mutants, which are defective in the processing of DSBs.

### Radiation exposures less than 1 krad can increase translocation frequencies in *rad9, rad51,* and *rad51rad9* diploids

**FIGURE 4:**
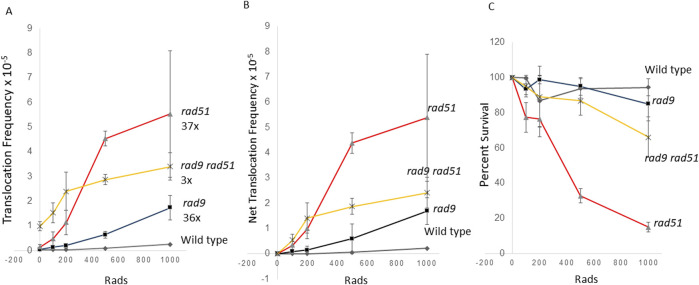
Stimulation of homology-directed translocations by “low dose” ionizing radiation in wild-type, *rad9, rad51,* and *rad9 rad51* diploid strains. Radiation-associated recombination frequencies (translocations) were measured after wild type, *rad51* (N = 3), *rad9* (N = 3) and *rad9 rad51* (N = 3) diploid strains were exposed to 100, 200, 500, 1,000 rads X rays. Recombination frequencies **(A)** were plotted against radiation dose. Net recombination frequencies were calculated by subtracting the spontaneous frequency from the radiation-associated frequency for each experiment **(B)**. The average survival percentage was plotted against radiation dose **(C)**. Filled triangle is the *rad51* strain (YB170), filled box is the *rad9* strain (YB134), filled diamond is the wild-type strain (YB110), and star is *rad9 rad51* (YB749). Fold change was calculated by dividing 1 krad-associated frequency by the spontaneous frequency in **(A)**.

We previously observed that the frequencies of radiation-associated rearrangements increase in *rad51* (Fasullo et al., 2004) and *rad9* mutants (Fasullo et al., 1998), defective in homologous recombination and G_2_ checkpoint control, respectively. However, it was unclear whether radiation-associated translocations could be detected at radiation exposures less than 1 krad (10 Gy); on average, 0.58 DSBs are introduced per krad in a diploid yeast cell (Frankenberg-Schwager and Frankenberg, 1990; Westmoreland and Resnick, 2016). We therefore determined whether *rad9*, *rad51*, and *rad9 rad51* diploid mutants exhibit higher levels of radiation-associated translocations frequencies after exposures of less than 1 krad. We measured translocations frequencies after exposure to 1 krad, 500 rads, 200 rads and 100 rads of X-radiation (Figure 4). While after 1 krad X-ray exposure we observed a seven-fold increase in the frequencies of translocations in wild type diploids, we observed a 36-fold and 47-fold increase in radiation-associated frequencies in *rad9* and *rad51* mutants, respectively, but only a three-fold increase in the frequencies of translocations in the double *rad9 rad51* double mutant. However, in the Rad^+^ diploid we did not observe higher levels radiation-associated frequences after cells were exposed to lower levels of radiation (Figure 4). Collectively, frequencies of radiation-associated translocations were significantly different in *rad9*, *rad51*, *rad9 rad51* after radiation exposures at all doses, compared to frequencies of spontaneous translocations (ANOVA, P < 0.05); radiation-associated frequencies in *rad9*, *rad51*, and *rad9 rad51* mutants after exposure to 500 rad were all significantly different than frequencies of spontaneous translocation. *rad9 rad51* mutant showed the highest dose-associated significance after 200 rad (Dunnett’s, P < 0.05), 500 rad (Dunnett’s, P < 0.01, and 1 krad (Dunnett’s, P < 0.001), compared to the other strains. Compared to net frequencies of translocations after 100 rad exposures in wild-type cells, those obtained in *rad51* (Dunnett’s, P < 0.04) and in *rad51 rad9* cells (Dunnett’s, P < 0.003) were significantly different. Interestingly, the *rad9 rad51* diploid mutant was more resistant to ionizing radiation at low radiation doses than the *rad51* mutant. We speculate that the *rad9 rad51* diploid mutant may be able to tolerate low levels of radiation than *rad51* mutant due to lack of checkpoint function. On the other hand, we speculate that His^+^ recombinants generated in *rad9 rad51* mutants after exposure to higher levels of radiation may contain more chromosomal rearrangements or chromosomal loss events, which may limit their viability. These studies thus indicate that frequencies of radiation-associated rearrangements increase in yeast mutants defective in DSB repair at exposures to low doses (100 rads) of ionizing radiation.

## Discussion

DNA repair and cell cycle checkpoint mutants exhibit higher frequencies of spontaneous chromosomal rearrangements. While higher frequencies of GCRs have been well-documented in *rad* and checkpoint yeast mutants (Myung et al., 2004; Smith et al., 2004), fewer studies have been performed regarding spontaneous and DNA damage-associated translocations generated by recombination between small repeat sequences, similar in size to human Alu sequences. We previously documented that higher frequencies of spontaneous and DNA damage associated translocations occur in *rad51* (Fasullo et al., 2001), *rad9* checkpoint mutants (Fasullo et al., 1998), and in *mec1-21* hypomorphic mutants defective in the S phase checkpoint (Fasullo et al., 2010). In this study, we determined whether the *RAD51*-mediated recombination pathway, the *RAD9*-mediated checkpoint pathway, and the S phase checkpoint pathway are independent pathways in suppressing homology directed translocations. Our overall conclusion is that knocking out functions in both the *RAD9* checkpoint and DSB repair pathways confers a synergistic increase in the rates of spontaneous translocations, while knocking down *MEC1*-mediated S phase checkpoint function and DSB repair conferred the highest frequencies of spontaneous translocations. In turn, specific *rad* mutants exhibit higher frequencies of radiation-associated translocations, even at relative low X-ray radiation exposures (100 rads). These studies are the first studies to document that radiation can stimulate homology-directed translocations in mutants defective in the MRX complex. We discuss our conclusions in the context of ploidy, comparisons with other studies performed in yeast, and implications for future studies in mammalian organisms.

We based our conclusions on results obtained by measuring translocation frequencies in (a/α) diploid mutants containing *his3* recombinational substrates positioned on chromosomes II and IV, containing 300 bp of continuous sequence similarity. The advantage to measuring homology-directed translocations in diploid strains is that *MAT* heterozygosity suppresses non-homologous end joining by repressing *NEJI* (Kegel et al., 2001) and enhances DSB repair by homologous recombination (Fasullo and Dave, 1994). In addition, diploid His^+^ recombinants containing either non-reciprocal or reciprocal translocations are viable; haploids containing non-reciprocal translocations would not be viable due to the elimination of essential genes. However, frequencies of His^+^ recombinants generated by recombination between *his3* truncated fragments only measure a subset of possible outcomes of recombination between short repeated sequences; for example, gene conversion events are not detected. Previous characterization of electrophoretic karyotypes exhibited by *rad9* (Fasullo et al., 1998) and *rad51* mutants (Fasullo et al., 2001) suggest that non-reciprocal and additional chromosomal rearrangements besides the directed translocations are also present and are currently under investigation.

Our results may initially seem to contradict results indicating that yeast ploidy decreases genetic instability (Tourrette et al., 2007). This study used a *ura2 15-30-72* mutated allele construct in which Ura^+^ revertants contain complex rearrangements or mutations. Although the frequency of Ura^+^ revertants are decreased in diploid strains, there is an enrichment of Ura^+^ revertants that contain non-reciprocal translocations. Thus, while the frequencies of specific types of genetic instabilities may decrease with an increase in ploidy, it may also increase frequencies of genome rearrangements that would potentially confer lethality in haploids and are thus consistent with our studies. Since mammalian cells are diploid and cancer cells can be polyploid, diploid cells may represent a better understanding of genetic instability phenotypes (Putnam and Kolodner, 2017).

Our studies support the idea that *RAD52* epistasis genes and checkpoint genes are independent pathways that ensure that sister chromatids are preferred templates for DSB repair, and when these functions are defective, there is a higher frequency that repeated sequences present on non-homologous chromosomes are utilized for recombinational repair. An attractive notion is that while *RAD9* may delay the cell cycle to allow sufficient time for sister chromatid recombination (Fasullo et al., 1998; Mathiasen and Lisby, 2014), *RAD51* promotes damage-induced sister chromatid recombination and suppresses alternative recombination pathways that generate half-reciprocal or reciprocal translocations. Thus, the synergistic increase in translocation frequency observed in *rad9 rad51* double mutants may result from the persistence and accumulation of chromosomal fragments and the elevation of alternative pathways for repair of DSBs, such as single-strand annealing (SSA). While the precise mechanisms that generate non-reciprocal and reciprocal translocations in *rad51* and *rad9* mutants are unknown, translocations can be generated by break-induced replication (BIR, Sakofsky and Malkova, 2017) and SSA (Manthey and Bailis, 2010). BIR can also occur in *rad51* mutants but requires *RAD50* (Signon et al., 2001). SSA is an attractive mechanism since it is suppressed by *RAD51* (Gallagher et al., 2020) and requires *RAD59* (Pannunzio et al., 2012), which was required for the elevated rate of translocations observed in the *rad51* diploid (Table 1). Deficient inhibition of Sgs1 in *rad9* mutants (Ferrari et al., 2020) and *de novo* telomere formation (Marcomini et al., 2018) could also result in non-reciprocal chromosomal rearrangements. Thus, further characterization of chromosomal rearrangements in *rad9 rad51* mutants is required to elucidate possible mechanisms.

Our surprising result was that *mre11, rad50, xrs2* mutants higher frequencies of radiation-associated translocations and that *rad9 mre11* and *rad9 rad50* mutants exhibited synergistic increases in rate of spontaneous translocation, considering that MRX genes are required for the processing of DSBs and precede *RAD9* in the checkpoint response to DSBs (Usui et al., 2001). Previous studies have indicated that redundant nucleases may resect the ends of DSBs (for reviews see, Mimitou and Symington, 2009; Cejka and Symington, 2021), among which are Mre11, Exo1 and Dna2, in cooperation with the Sgs1 helicase (Zhu et al., 2008). Our studies thus provoke questions as to which nucleases function in generating the recombinogenic 3′ ends that are required for homology-directed translocations. However, Westmoreland and Resnick (2016) demonstrate that radiation-associated cross-over events occur in mutants that exhibit little resection and Ivanov et al. (1994), observed that mating type switching occurs in *xrs2* and *rad50* mutants. In addition, Marcomini et al. (2018) observed that MRX deficiencies lead to greater mobility of chromosomal fragments, which could facilitate the formation of chromosomal rearrangements. Our data thus underscore that MRX complex suppresses ectopic recombination between repeated sequences (Oh and Symington, 2018).

The higher rates of spontaneous translocations in MRX mutants are consistent with studies that have shown that both *rad50* (Malone et al., 1990) and *mre11* (Bressan et al., 1999) mutants exhibit higher rates of heteroallelic recombination and cross-overs. Interestingly, *rad50 rad9* double mutants did not exhibit enhanced rates of spontaneous translocation, compared to rates observed in the *rad50* mutant, while *mre11 rad9* and *xrs2 rad9* double mutants do exhibit enhanced rates, compared to the single mutants (Table 1). Although the exact reason for differences in recombination phenotypes are unknown, previous studies have shown that *mms2 rad50* and *mms2 xrs2* double mutants exhibit different UV sensitivities, compared to the single mutants (Gangavarapu et al., 2007). In addition, Hailemariam et al. (2019), demonstrated that *RAD50* but not *MRE11*or *XRS2* was absolutely required for *TEL1* activation. Thus the genetic instability phenotypes of *rad50* may be different than that of *xrs2* or *mre11*.

**FIGURE 5:**
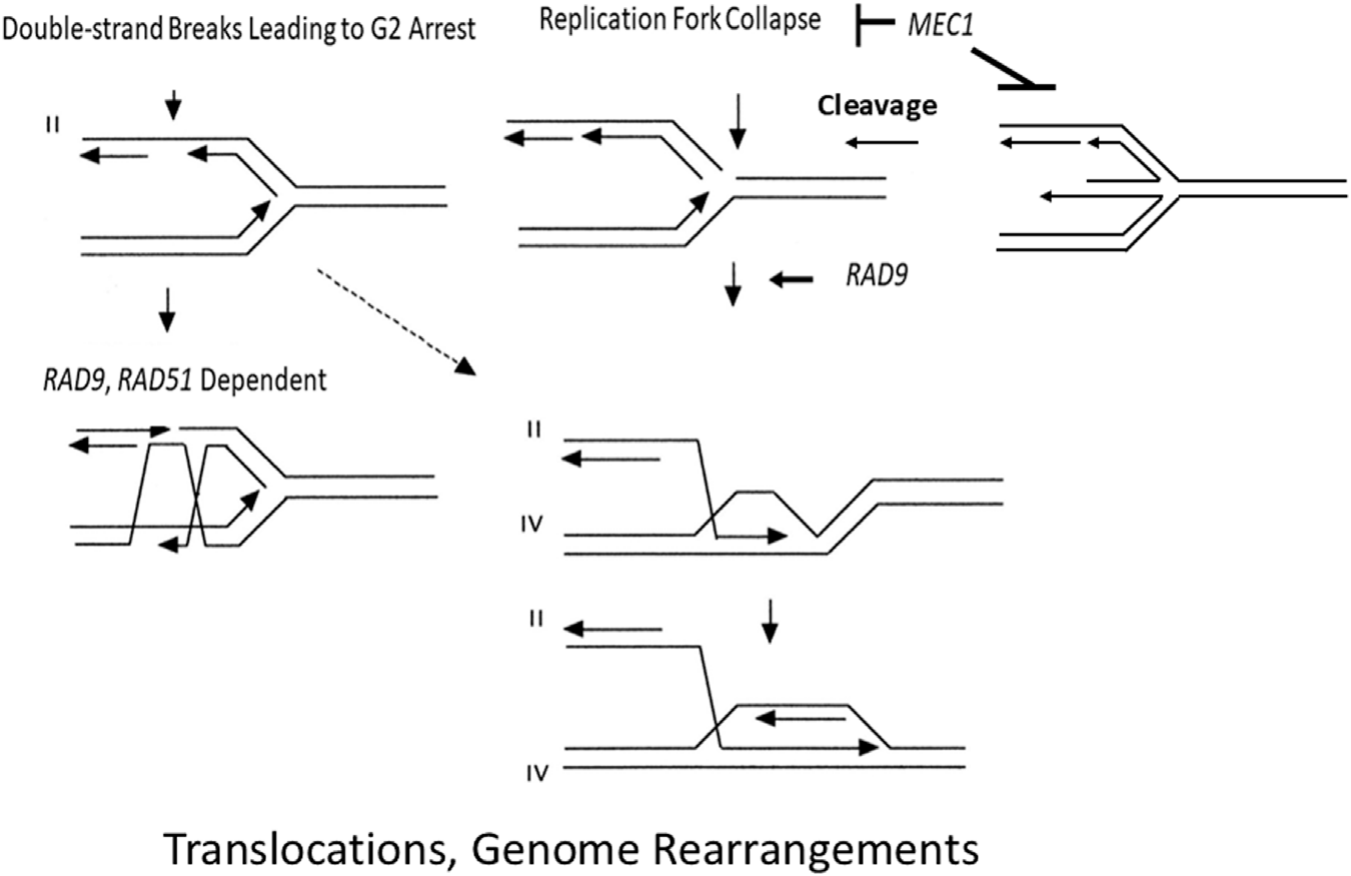
A proposed model of how defective gap repair in *rad51* and *rad9* mutants could enhance ectopic recombination between *his3* fragments, resulting in the generation of a nonreciprocal translocation involving chromosomes II and IV. Each line represents a single strand of DNA, and the arrow indicates the 3′end. Chromosomes II and IV are designated by roman numerals. (Left) The DSB occurs at a site on the sister chromatid after polymerase progression and initiates SCE by gap repair. *RAD51* and *RAD9* function in redundant pathways to promote recombinational repair and suppress ectopic recombination. (Right) The DSB occurs because of a collapsed replication fork are after replication fork regression. Replication is then reinitiated after the 3′ end of the broken chromatid invades the intact sister chromatid. BIR can generate non-reciprocal translocations if the 3′ end of the broken chromatid invades an intact nonhomologous chromosome. The dashed arrow indicates the possibility that a DSB, which cannot be repaired by gap repair in a *rad51* mutant, can initiate BIR and generate a nonreciprocal translocation. DSBs occurring in S phase require *RAD9* function to efficiently initiate repair of collapsed forks. The figure was adapted from Fasullo et al. (2010).

In agreement with the genetic control of GCRs (Pennaneach and Kolodner, 2004), our results indicate that the S phase checkpoint and *RAD51* participate in independent pathways for suppressing chromosomal rearrangements (Figure 5). We speculate that lesions that initiate homology-directed translocations in *mec1* mutants are generated by DNA replication fork collapse and that *rad51* mutants are defective in replication fork maintenance and replication restart. Our data indicate that such lesions also stimulate recombination between sister chromatids and homologs (Table 2). *RAD51* may also function to maintain DNA replication forks by non-recombination mechanisms, such as translesion DNA synthesis (Cano-Linares et al., 2021). However, in contrast to genetic control of GCRs, we previously observed that the highest frequencies of rearrangements occur in the *mec1-21* mutant, which retains some G_2_ checkpoint function, and not the *mec1* null mutant, which is defective in both S phase and G_2_ checkpoint function (Fasullo and Sun, 2008b). In our studies, the *mec1-21 rad9* double mutant exhibits lower rates of translocations, compared to *mec-21* (Fasullo and Sun, 2008a). Thus, we suggest that *RAD9*-mediated cell cycle delay is required to some of the recombination events that are initiated when replication fork collapse (Figure 5).

The additional checkpoint mutants we constructed are particularly useful in detecting the recombinogenic effects of low dose exposure to genotoxic agents, as previously observed for the *rad9* mutant (Fasullo et al., 2004). For example, relatively low doses of X rays (100 cGy) can stimulate chromosomal rearrangements in the *rad9 rad51* diploid mutant. This dose generates less than one DSB per diploid genome but would be sufficient to generate at least one single-strand break (Roots et al., 1990). The threshold dose for detecting radiation effects in budding yeast has yet to be determined. We suggest that checkpoint mutants containing the recombination substrates would also be useful in detecting genotoxic damage by other agents, such as topoisomerase inhibitors (Fasullo et al., 2004), which are known to cause replication fork collapse or DSBs.

Our observations have relevance to Alu recombination in mammalian cells. DSBs targeted to Alu sequences have been shown to initiate translocations by SSA mechanisms (Elliott et al., 2005). The observation that knocking out *RAD51* paralogs also confers genetic instability in yeast raises the question of whether deficiencies in other yeast or mammalian Rad51 paralogs (Liu et al., 1998) also confers higher frequencies of homology-directed rearrangements (Morales et al., 2021). Such studies may elucidate the role of polymorphisms in human X-ray repair genes (XRCC) associated with cancer (Liu et al., 2023).

In conclusion, we identified genes in independent pathways that suppress homology-directed translocations. These pathways underscore the role of the RAD9-mediated checkpoint pathway in suppressing homology-directed translocations and in enhancing recombination when the S phase checkpoint fail. It will be interesting to identify whether similar independent pathways suppress recombination between human Alu repeats.

## Data Availability

The original contributions presented in the study are included in the article/Supplementary Material, further inquiries can be directed to the corresponding author.

## Supplementary material

SUPPLEMENTARY FIGURE S1 Radiation sensitivities of wild type, *rad9*, *xrs2*, *rad9 xrs2*, *rad9 rad50*, *mre11*, and *rad9 mre11* diploid strains. After each strain was cultured in 2 mL of YPD, cells were serially diluted by 10^−2^ and 10^−4^ fold. 5 μL aliquots were then inoculated onto YPD plates. Plated were then either exposed to no radiation, 6 krad X rays, 60 J/m^2^ UV. Plates were incubated at 30°C for 3 days and then imaged.
