# Supplementary material for: Checkpoint and recombination pathways independently suppress rates of spontaneous homology-directed chromosomal translocations in budding yeast
Source: Front Genet. 2025 Apr 4;16:1479307. doi: 10.3389/fgene.2025.1479307 (PMC12006765; doi:10.3389/fgene.2025.1479307)
Supplement: Supplementary file 1 [file Table1.docx]

SUPPLEMENTARY TABLE 1. Yeast Strains

| Strain | Mating Type | Genotype | Derivation (Source) | Reference |
| --- | --- | --- | --- | --- |
| BY4741 | *MAT***a** | *MAT***a** *his3Δ1 leu2Δ0 met15Δ0 ura3Δ0* | Yeast Consortium | Brachman *et al.* (1998) |
| YA188 | *MAT***a** | *MAT***a** *his3Δ1 leu2Δ0 met15Δ0 ura3Δ0 rad59::KanMX* | Resgen company (3756) |  |
| FY250 | *MAT*α | *ura3-52, his3- Δ200, trp1-Δ63, leu2-Δ1* | F. Winston | Winston *et. al.* (1995) |
| FY251 | *MAT***a** | *ura3-52, his3- Δ200, trp1- Δ63, leu2-Δ1,* | F. Winston | Winston *et. al.* (1995) |
| YA102 | *MAT***a** | *ura3-52 his3- Δ200 ade2-101 lys2-801 trp1- Δ1 gal3-* | M. Carlson |  |
| YA148 | *MAT***a** | *ura3-52,his3- Δ200,ade2-101,lys2-801,leu2- Δ1,trp1- Δ63, cup1::ura3* | C. Guthrie | Lesser and Guthrie. (1994) |
| YA149 | *MAT*α | *ura3-52,his3- Δ200,ade2-101,lys2-801,leu2- Δ1,trp1- Δ63, cup1::ura3* | C. Guthrie | Lesser and Guthrie, (1994) |
| YB132 | *MAT***a** | *ura3-52 his3- Δ200 ade2-101 lys2-801 trp1-Δ1 gal3 rad9::URA3* | *rad9::URA3* disuption in YA102 | Fasullo *et al*., 1998 |
| YB315 | *MAT***a** | *ura3-52 his3- Δ200 ade2-a lys2-801 trp1-Δ1 gal3* | This Lab | Fasullo *et al*., 2010 |
| YB316 | *MAT***a** | *ura3-52 his3- Δ200 ade2-a lys2-801 trp1- Δ 1 gal3 mec1-21* | This laboratory | Fasullo *et al*., 2010 |
| YB698 | *MAT***a** | *ura3-52 his3-Δ200 ade2-a lys2-801 trp1-Δ1 gal3 rad9::URA3* | *rad9::URA3* disuption in YB315 | This work |
| YB699 | *MAT***a** | *ura3-52 his3- Δ200 ade2-a leu2-Δ1 trp1-Δ1 gal3- rad9::URA3* | Meiotic segregant of genetic cross of YB315 x FY250 | This work |
| YB132 | *MAT***a** | *ura3-52 his3- Δ200 ade2-101 lys2-801 trp1-Δ1 gal3 rad9::URA3* | This laboratory | Fasullo *et al*. (1998) |
| YB133 | *MAT***a** | *ura3-52,his3- Δ200,ade2-101,lys2-801,leu2- Δ 1,trp1- Δ63, cup1::ura3*  *rad9::LEU2* | *rad9::LEU2* in YA148 | Fasullo *et al*., 1998 |
| YB700 | *MAT***a** | *ura3-52 his3- Δ200 ade2-a lys2-801 trp1-Δ1 gal3 rad51::URA3* | *rad51::URA3* disruption in YB315 | This work |
| YB701 | *MAT***a** | *ura3-52 his3- Δ200 ade2-a lys2-801 trp1-Δ 1 gal3 mec1-21 rad9::URA3* | *rad9::URA3* disruption in YB316 | This work |
| YB702 | *MAT***a** | *ura3-52 his3- Δ200 ade2-a lys2-801 trp1- Δ 1 gal3 mec1-21 rad51::URA3* | *rad51::URA3* disruption in YB316 | This work |
| YB169 | *MAT***a** | *ura3-52 his3- Δ200 ade2-101 lys2-801 trp1- Δ1 gal3 rad51::URA3* | *rad51:URA3* disruption in YA102 | Fasullo *et al*. (2001) |
| YB703 | *MAT***a** | *ura3-52, his3-Δ200, trp1-Δ 63, leu2- Δ 1, rad9::LEU2* | *rad9::LEU2 disruption in* FY251 | This work |
| YB704 | *MAT***a** | *ura3-52, his3-Δ200, trp1-Δ 63, leu2-Δ 1,*  *rad55::LEU2* | *rad55::LEU2* disruption in FY251 | This work |
| YB705 | *MAT***a** | *ura3-52, his3-Δ200, trp1-Δ63, leu2-Δ 1, rad57::LEU2* | *rad57::LEU2* disruption in FY251 | This work |
| YB706 | *MAT***a** | *ura3-52, his3- Δ200, trp1-Δ63, leu2-Δ 1, rad54::LEU2* | *rad54::LEU2* disruption in FY251 | This work |
| YB707 | *MAT***a** | *ura3-52 his3- Δ200 ade2-a lys2-801 trp1-Δ1 gal3-* | *mre11:URA3* disruption in YB135 | This work |
| YB708 | *MAT***a** | *ura3-52 his3- Δ200 ade2-101 lys2-801 trp1- Δ 1 gal3 rad50::URA3* | *rad50:URA3* disruption in YA102 | This work |
| YB709 | *MAT***a** | *ura3-52, his3-Δ200, trp1- Δ 63, leu2-Δ1, ade2-101, xrs2::LEU2* | *xrs2::LEU2* disruption in FY251 | This work |
| YB710 | *MAT***a** | *ura3-52, his3-Δ200, trp1- Δ63, leu2- Δ 1, ade2-101, rad54::URA3* | *rad54::URA3* disruption in YA102 | This work |
| YB711 | *MAT***a** | *ura3-52 his3- Δ 200 ade2-a lys2-801 trp1- Δ 1 gal3- rad59:kanMX* | *rad59::kanMX* disruption in YB315 | This work |
| YB712 | *MAT***a** | *ura3-52,his3- Δ 200,ade2-101,lys2-801,leu2- Δ 1,trp1- Δ 63, cup1::ura3*  *rad9::LEU2 rad51::URA3* | *rad51::URA3* disruption in YB133 | This work |
| YB713 | *MAT***a** | *ura3-52 his3- Δ200 , leu2-Δ ade2-101 trp1 rad9::URA3 rad55::LEU2* | *rad55::LEU2 disruption in* YB699 | This work |
| YB714 | *MAT***a** | *ura3-52, his3-Δ200, trp1-Δ 63, leu2- Δ 1, ade2-101 rad9::URA3 rad57::LEU2* | *rad57::LEU2* disruption in YB699 | This work |
| YB715 | *MAT***a** | *ura3-52,his3- Δ 200,ade2-101,lys2-801,leu2- Δ 1,trp1- Δ 63, cup1::ura3*  *rad9::LEU2 rad54::URA3* | *rad54::URA3* disruption in  YB133 | This work |
| YB716 | *MAT***a** | *ura3-52,his3- Δ 200,ade2-101,lys2-801,leu2- Δ 1,trp1- Δ 63, cup1::ura3*  *rad9::LEU2 mre11::URA3* | *mre11::URA3* disruption in YB133 | This work |
| YB717 | *MAT***a** | *ura3-52,his3- Δ200,ade2-101,lys2-801,leu2- Δ1,trp1- Δ63, cup1::ura3*  *rad9::LEU2 rad50::URA3* | *rad50*::*URA3* disruption in  YB133 | This work |
| YB718 | *MAT***a** | *ura3-52, his3-Δ200, trp1-Δ 63, leu2- Δ 1, ade2-101 rad9::URA3 xrs2::LEU2* | *xrs2::LEU2* disruption in YB699 | This work |
| YB719 | *MAT***a** | *ura3-52, his3-Δ200, ade2-a, lys2-801, trp1- Δ1, gal3- rad59:kanMX rad51::URA3* | *rad51::URA3* disruption in YB711 | This work |
| YB760 | *MAT***a** | *ura3-52, his3-Δ200, ade2-a, lys2-801, trp1- Δ1, gal3- rad59:kanMX rad9::URA3* | *rad9::URA3* disruption in YB711 | This work |
| Haploid strains used to measure homology-directed translocations, | | | | |
| YB109 | *MAT*α | *ura3-52,his3- Δ 200,ade2-101, leu2-3, 112 lys2-801,trp1- Δ 1 gal3, GAL1::his3- Δ 5',trp1::his3-Δ 3'::HOcs* | This lab | Fasullo *et al.* (1998) |
| YB318 | *MAT*α | *ura3-52, his3- Δ200, ade2-n ,lys2-801, trp1-Δ1 gal3, GAL1::his3-Δ5', trp1::his3- Δ 3'::HOcs* | This lab | Fasullo *et al*. (2010) |
| YB319 | *MAT*α | *ura3-52, his3- Δ 200, ade2-n, trp1-1Δ*, *gal3, leu2-3, 112 GAL1::his3-5′*  *trp1::his3- Δ 3′ ::HOcs lys2(leaky) mec1-21* | This lab | Fasullo *et al.* (2010) |
| YB130 | *MAT*α | *ura3-52,his3- Δ200,ade2-101,lys2-801,trp1-Δ1, gal3, leu2-3, 112, GAL1::his3- Δ 5',trp1::his3- Δ 3'::HOcs, rad9::URA3* | *rad9::URA3* disruption in YB109 | Fasullo *et al.*(1998) |
| YB131 | *MAT*α | *ura3-52,his3- Δ200,ade2-101,lys2-801,trp1- Δ1, leu2-3, 112, gal3, GAL1::his3-Δ5',trp1::his3-Δ 3'::HOcs, rad9::LEU2* | *rad9::LEU2* disruption in YB109 | Fasullo *et al.*(1998) |
| YB720 | *MAT*α | *ura3-52,his3- Δ200,ade2-n,lys2-801,trp1- Δ1 gal3, GAL1::his3-Δ 5',trp1::his3- Δ 3'::HOcs, rad9::URA3* | *rad9::URA3* disruption in YB318 |  |
| YB168 | *MAT*α | *ura3-52,his3- Δ 200,ade2-101,lys2-801,trp1- Δ 1gal3, GAL1::his3-Δ 5',trp1::his3- Δ 3'::HOcs, rad51:URA3* | *rad51:URA3* disruption in YB109 | Fasullo *et al*, 2010 |
| YB721 | *MAT*α | *ura3-52,his3- Δ200,ade2-n,lys2-801,trp1- Δ1 gal3, GAL1::his3-Δ 5',trp1::his3- Δ 3'::HOcs, rad51::URA3* | *rad51::URA3* disruption in YB318 |  |
| YB722 | *MAT*α | *ura3-52,his3- Δ 200,ade2-101,lys2-801,trp1- Δ 1 gal3, leu2-3, 112, GAL1::his3-D5',trp1::his3- Δ3'::HOcs,*  *rad55::LEU2* | *rad55::LEU2* disruption in YB109 | This work |
| YB723 | *MAT*α | *ura3-52,his3-Δ200,ade2-101,lys2-801, trp1- Δ1 gal3, leu2-3, 112, GAL1::his3-Δ 5',trp1::his3-Δ3'::HOcs, rad57::LEU2* | *rad57::LEU2* disruption in YB109 | This work |
| YB724 | *MAT*α | *ura3-52,his3-Δ200,ade2-101,lys2-801, trp1- Δ1 gal3, leu2-3, 112, GAL1::his3-Δ 5',trp1::his3-Δ3'::HOcs, rad54::URA3* | *rad54::URA3* disruption in YB109 | This work |
| YB725 | *MAT*α | *ura3-52,his3- Δ200,ade2-101,lys2-801,trp1- Δ 1gal3, leu2-3, 112, GAL1::his3-Δ5',trp1::his3-Δ 3'::HOcs,*  *mre11:URA3* | *mre11:URA3* disruption in YB109 | This work |
| YB726 | *MAT*α | *ura3-52,his3-Δ200,ade2-101,lys2-801, trp1- Δ1 gal3, GAL1::his3-Δ 5',trp1::his3-Δ3'::HOcs, rad50:URA3* | *rad50:URA3* disruption in YB109 | This work |
| YB727 | *MAT*α | *ura3-52,his3-Δ200,ade2-101,lys2-801, trp1- Δ1 gal3, GAL1::his3-Δ 5',trp1::his3-Δ3'::HOcs, xrs2::LEU2* | *xrs2::LEU2* disruption in YB109 | This work |
| YB728 | *MAT*α | *ura3-52,his3-Δ200,ade2-101,lys2-801, trp1- Δ1 gal3, GAL1::his3-Δ 5',trp1::his3-Δ3'::HOcs, rad59::kanMX* | *rad59::kanMX* disruption in YB109 | This work |
| YB729 | *MAT*α | *ura3-52,his3- Δ200,ade2-101,lys2-801,trp1- Δ 1gal3, GAL1::his3-Δ5',trp1::his3-Δ 3'::HOcs, rad9::LEU2 rad51::URA3* | *rad51* disruption in YB131 | This work |
| YB730 | *MAT*α | *ura3-52,his3- Δ200,ade2-101,lys2-801,trp1- Δ 1gal3, GAL1::his3-Δ5',trp1::his3-Δ 3'::HOcs, rad9::URA3 rad55::LEU2* | *rad55* disruption in YB130 | This work |
| YB731 | *MAT*α | *ura3-52,his3- Δ200,ade2-101,lys2-801,trp1- Δ 1gal3, GAL1::his3-Δ5',trp1::his3-Δ 3'::HOcs, rad9::URA3 rad57::LEU2* | *rad57* disruption in YB130 | This work |
| YB732 | *MAT*α | *ura3-52,his3- Δ200,ade2-101,lys2-801,trp1- Δ 1gal3, GAL1::his3-Δ5',trp1::his3-Δ 3'::HOcs, rad9::LEU2 rad54::URA3* | *rad54* disruption in YB131 | This work |
| YB733 | *MAT*α | *ura3-52,his3- Δ200,ade2-101,lys2-801,trp1- Δ 1gal3, GAL1::his3-Δ5',trp1::his3-Δ 3'::HOcs, rad9::LEU2 mre11::URA3* | *mre11* disruption in YB131 | This work |
| YB734 | *MAT*α | *ura3-52, his3-Δ200, ade2-101, lys2-801, trp1-Δ1 gal3, GAL1::his3-Δ5',trp1::his3-Δ 3'::HOcs, rad9::LEU2 rad50::URA3* | *rad50* disruption in YB131 | This work |
| YB735 | *MAT*α | *ura3-52,his3- Δ200,ade2-101,lys2-801,trp1- Δ 1gal3, GAL1::his3-Δ5',trp1::his3-Δ 3'::HOcs, rad9::URA3 xrs2::LEU2* | *xrs2* disruption in YB130 | This work |
| YB736 | *MAT*α | *ura3-52,his3- Δ200,ade2-101,lys2-801,trp1- Δ 1gal3, GAL1::his3-Δ5',trp1::his3-Δ 3'::HOcs, rad51::URA3, rad59::kanMX* | *rad51* disruption in YB728 | This work |
| YB737 | *MAT*α | *ura3-52, his3- Δ 200, ade2-n, trp1-1Δ*, *gal3, leu2-3, 112 GAL1::his3-5′*  *trp1::his3- Δ 3′ ::HOcs lys2(leaky) mec1-21, rad51:URA3* | *rad51* disruption in YB319 | This work |
| YB738 | *MAT*α | *ura3-52, his3- Δ 200, ade2-n, trp1-1Δ*, *gal3, leu2-3, 112 GAL1::his3-5′*  *trp1::his3- Δ 3′ ::HOcs lys2(leaky) mec1-21, rad9:URA3* | *rad9* disruption in YB319 | This work |
| YB761 | *MATα* | *ura3-52,his3- Δ200,ade2-101,lys2-801,trp1- Δ 1gal3, GAL1::his3-Δ5',trp1::his3-Δ 3'::HOcs, rad9::URA3, rad59::kanMX* | *rad9* disruption in YB728 | This work |
| Diploid strains used to monitor homology-directed translocations | | | | |
| YB110 | *MAT***a***/MAT*α | *ura3-52/-, his3-Δ200/- , trp1-Δ 1/-, ade2-101/- , lys2-801/-, leu2-3, 112/LEU2, GAL1::his3-Δ5',trp1::his3-Δ 3'::Hocs* | Diploid cross YB109 x YA102 | Fasullo *et al.*(1998) |
| YB348 | *MAT***a***/MAT*α | *ura3-52/-, his3-Δ200/- , trp1-Δ1/-, ade2-ade2-a/ade2-n , lys2-801/lys2-801, leu2-3, 112/LEU2, GAL1::his3-Δ5',trp1::his3-Δ 3'::HOcs* | Diploid cross YB315 x YB318 | Fasullo *et al*. (2010) |
| YB134 | *MAT***a***/MAT*α | *ura3-52/-, his3-Δ200/- , trp1-Δ 1/-, ade2-101/- , lys2-801/-, leu2-3, 112/LEU2, GAL1::his3-Δ5',trp1::his3-Δ 3'::HOcs rad9::URA3/rad9::URA3* | Diploid cross YB130 x YB132 | Fasullo *et al.*(1998) |
| YB135 | *MAT***a***/MAT*α | *ura3-52/-*,  *his3-Δ200/-*,  *ade2-101/- lys2-801/-* , *leu2-Δ1/ leu2-3, 112, trp1-Δ63/ trp1- Δ1 Δcup1::ura3, GAL1::his3-Δ5',trp1::his3-Δ 3'::HOcs,*  *rad9::LEU2/rad9::LEU2* | Diploid cross YB131 x YB133 | Fasullo *et al.*(1998) |
| YB740 | *MAT***a***/MAT*α | *ura3-52/-*,  *his3-Δ200/-*,  *ade2-a/ade2-n lys2-801/LYS2* , *leu2-Δ1/ leu2-3, 112, trp1-Δ1/ trp1- Δ1 GAL1::his3-Δ5',trp1::his3-Δ 3'::HOcs, rad9::URA3/rad9::URA3* | Diploid cross of YB698 x YB720 | This work |
| YB170 | *MAT***a***/MAT*α | *ura3-52/-, his3-Δ200/- , trp1-Δ 1/-, ade2-101/- , lys2-801/-, leu2-3, 112/LEU2, GAL1::his3-Δ5',trp1::his3-Δ 3'::HOcs rad51::URA3/rad51::URA3* | Diploid cross of YB168 x  YB169 | Fasullo *et al*. (2010) |
| YB741 | *MAT***a***/MAT*α | *ura3-52/-, his3-Δ200/- , trp1-Δ 1/-, ade2-101/- , lys2-801/-, leu2-3, 112/LEU2, GAL1::his3-Δ5',trp1::his3-Δ 3'::HOcs rad54::URA3/rad54::URA3* | Diploid cross of YB710 x YB724 | Fasullo *et al*. (2010) |
| YB742 | *MAT***a***/MAT*α | *ura3-52/-, his3-Δ200/- , trp1-Δ 1/-, ade2-a/ade2-n, lys2-801/-, leu2-3, 112/LEU2, GAL1::his3-Δ5',trp1:1:his3-Δ 3'::HOcs rad51::URA3/rad51::URA3* | Diploid cross of YB700 x YB721 | This work |
| YB743 | *MAT***a***/MAT*α | *ura3-52/-, his3-Δ200/- , trp1-Δ 1/-, ade2-101/- , lys2-801/-, leu2-3, 112/LEU2, GAL1::his3-Δ5',trp1::his3-Δ 3'::HOcs mre11::URA3/mre11::URA3* | Diploid cross of YB707 x YB725 | This work |
| YB744 | *MAT***a***/MAT*α | *ura3-52/-, his3-Δ200/- , trp1-Δ 1/-, ade2-101/- , lys2-801/LYS2, leu2-3, 112/leu2, GAL1::his3-Δ5',trp1::his3-Δ 3'::HOcs rad55::LEU2/rad55::LEU2* | Diploid cross of YB704 x YB722 | This work |
| YB745 | *MAT***a***/MAT*α | *ura3-52/-, his3-Δ200/- , trp1-Δ 1/-, ade2-101/- , lys2-801/LYS2, leu2-3, 112/leu2, GAL1::his3-Δ5',trp1::his3-Δ 3'::HOcs rad57::LEU2/rad57::LEU2* | Diploid cross of YB705 x YB723 | This work |
| YB746 | *MAT***a***/MAT*α | *ura3-52/-, his3-Δ200/- , trp1-Δ 1/-, ade2-101/- , lys2-801/-, leu2-3, 112/LEU2, GAL1::his3-Δ5',trp1::his3-Δ 3'::HOcs rad50::URA3/rad50::URA3* | Diploid cross of YB708 x YB726 | This work |
| YB747 | *MAT***a***/MAT*α | *ura3-52/-, his3-Δ200/- , trp1-Δ1/-, ade2-101/- , lys2-801/LYS2, leu2-3,112/leu2, GAL1::his3-Δ5',trp1::his3-Δ 3'::HOcs xrs2::LEU2/xrs2::LEU2* | Diploid cross of YB709 x YB727 | This work |
| YB748 | *MAT***a***/MAT*α | *ura3-52/-, his3-Δ200/- , trp1-Δ 1/-, ade2-101/- , lys2-801/-, leu2-3, 112/LEU2, GAL1::his3-Δ5',trp1::his3-Δ 3'::HOcs rad59::KanMX/rad59::KanMX* | Diploid cross of YB711 x YB728 | This work |
| YB749 | *MAT***a***/MAT*α | *ura3-52/-, his3-Δ200/- , trp1-Δ1/-, ade2-101/- , lys2-801/LYS2, leu2-3,112/leu2, GAL1::his3-Δ5',trp1::his3-Δ 3'::HOcs rad9::LEU2/rad9::LEU2 rad51::URA3/rad51::URA3* | Diploid cross of YB712 x YB729 | This work |
| YB750 | *MAT***a***/MAT*α | *ura3-52/-, his3-Δ200/- , trp1-Δ1/-, ade2-101/- , lys2-801/LYS2, leu2-3,112/leu2, GAL1::his3-Δ5',trp1::his3-Δ 3'::HOcs, rad9::LEU2/rad9::LEU2 , rad50::URA3/rad50::URA3* | Diploid cross of YB717 x YB734 | This work |
| YB751 | *MAT***a***/MAT*α | *ura3-52/-, his3-Δ200/- , trp1-Δ1/-, ade2-101/- , lys2-801/LYS2, leu2-3,112/leu2, GAL1::his3-Δ5',trp1::his3-Δ 3'::HOcs rad9::LEU2/rad9::LEU2 rad54::URA3/rad54::URA3* | Diploid cross of YB715 x YB732 | This work |
| YB752 | *MAT***a***/MAT*α | *ura3-52/-, his3-Δ200/- , trp1-Δ 1/-, ade2-101/- , lys2-801/-, leu2-3, 112/LEU2, GAL1::his3-Δ5',trp1::his3-Δ 3'::HOcs rad51::URA3/rad51::URA3 rad59::KanMX/rad59::KanMX* | Diploid cross of YB719 x YB736 | This work |
| YB753 | *MAT***a***/MAT*α | *ura3-52/-, his3-Δ200/- , trp1-Δ1/-, ade2-101/- , lys2-801/LYS2, leu2-3,112/leu2 Δ1, GAL1::his3-Δ5',trp1::his3-Δ 3'::HOcs rad9::URA3/rad9::URA3 rad55::LEU2/rad55::LEU2* | Diploid cross of YB713 x YB730 | This work |
| YB754 | *MAT***a***/MAT*α | *ura3-52/-, his3-Δ200/- , trp1-Δ1/-, ade2-101/- , lys2-801/LYS2, leu2-3,112/leu2 Δ1, GAL1::his3-Δ5',trp1::his3-Δ 3'::HOcs rad9::URA3/rad9::URA3 rad57::LEU2/rad57::LEU2* | Diploid cross of YB714 x YB731 | This work |
| YB755 | *MAT***a***/MAT*α | *ura3-52/-, his3-Δ200/- , trp1-Δ1/-, ade2-101/- , lys2-801/LYS2, leu2-3,112/leu2 Δ1, GAL1::his3-Δ5',trp1::his3-Δ 3'::HOcs rad9::URA3/rad9::URA3 xrs2::LEU2/xrs2::LEU2* | Diploid cross of YB718 x YB735 | This work |
| YB325 | *MAT***a***/MAT*α | *ura3-52/-, his3-Δ200/- , trp1-Δ1/-, ade2-a/ade2-n, lys2-801/LYS2, leu2-3,112/-, GAL1::his3-Δ5',trp1::his3-Δ 3'::HOcs,*  *mec1-21/mec1-21* | Diploid cross of YB316 x YB319 | Fasullo *et al.* (2010) |
| YB756 | *MAT***a***/MAT*α | *ura3-52/-, his3-Δ200/- , trp1-Δ1/-, ade2-a/ade2-n, lys2-801/LYS2, leu2-3,112/-, GAL1::his3-Δ5',trp1::his3-Δ 3'::HOcs,*  *mec1-21/mec1-21, rad9::URA3/rad9::URA3* | Diploid cross of YB701 x YB738 | This work |
| YB757 |  | *ura3-52/-, his3-Δ200/- , trp1-Δ1/-, ade2-a/ade2-n, lys2-801/LYS2, leu2-3,112/-, GAL1::his3-Δ5',trp1::his3-Δ 3'::HOcs,*  *mec1-21/mec1-21, rad51::URA3/rad51::URA3* | Diploid cross of YB702 x YB737 | This work |
| YB762 | *MAT***a***/MAT*α | *ura3-52/-, his3-Δ200/- , trp1-Δ 1/-, ade2-101/- , lys2-801/-, leu2-3, 112/LEU2, GAL1::his3-Δ5',trp1::his3-Δ 3'::HOcs rad9::URA3/rad9::URA3 rad59::KanMX/rad59::KanMX* | Diploid cross of YB760 x YB761 | This work |
| Strains to monitor sister-chromatid exchange | | | | |
|  |  |  |  |  |
| YB163 | *MAT***a-inc** | *ura3-52 his3- Δ200 ade2-101 lys2-801 trp1- Δ1 gal3 trp1::his3-Δ5’ his3-Δ3’* | This lab | Dong and Fasullo, (2003) |
| YB177 | *MAT***a-inc** | *ura3-52 his3- Δ200 ade2-101 lys2-801 trp1- Δ1 gal3 trp1::his3-Δ5’ his3-Δ3’* | This lab | Dong and Fasullo, (2003) |
| YB147 | *MAT***a-inc** | *ura3-52 his3- Δ200 ade2-101 lys2-801 trp1- Δ1 gal3 trp1::his3-Δ5’ his3-Δ3’ rad9::URA3* | *rad9::URA3* disruption | Fasullo et al., (1998) |
| YB311 | *MAT***a-inc** | *ura3-52 his3- Δ200 ade2-101 lys2-801 trp1- Δ1 gal3 trp1::his3-Δ5’ his3-Δ3’ mec1-21* | This lab | Fasullo *et al.* (2010) |
| YB758 | *MAT***a-inc** | *ura3-52 his3- Δ200 ade2-101 lys2-801 trp1- Δ1 gal3 trp1::his3-Δ5’ his3-Δ3’mec1-21 rad9* | *rad9::URA3* disruption in YB311 | This work |
| YB759 | *MAT***a-inc** | *ura3-52 his3- Δ200 ade2-101 lys2-801 trp1- Δ1 gal3 trp1::his3-Δ5’ his3-Δ3’mec1-21 rad51* | *rad51::URA3* disruption in YB311 | This work |
