# Supplementary figures and images for: Checkpoint and recombination pathways independently suppress rates of spontaneous homology-directed chromosomal translocations in budding yeast
Source: Front Genet. 2025 Apr 4;16:1479307. doi: 10.3389/fgene.2025.1479307 (PMC12006765; doi:10.3389/fgene.2025.1479307)

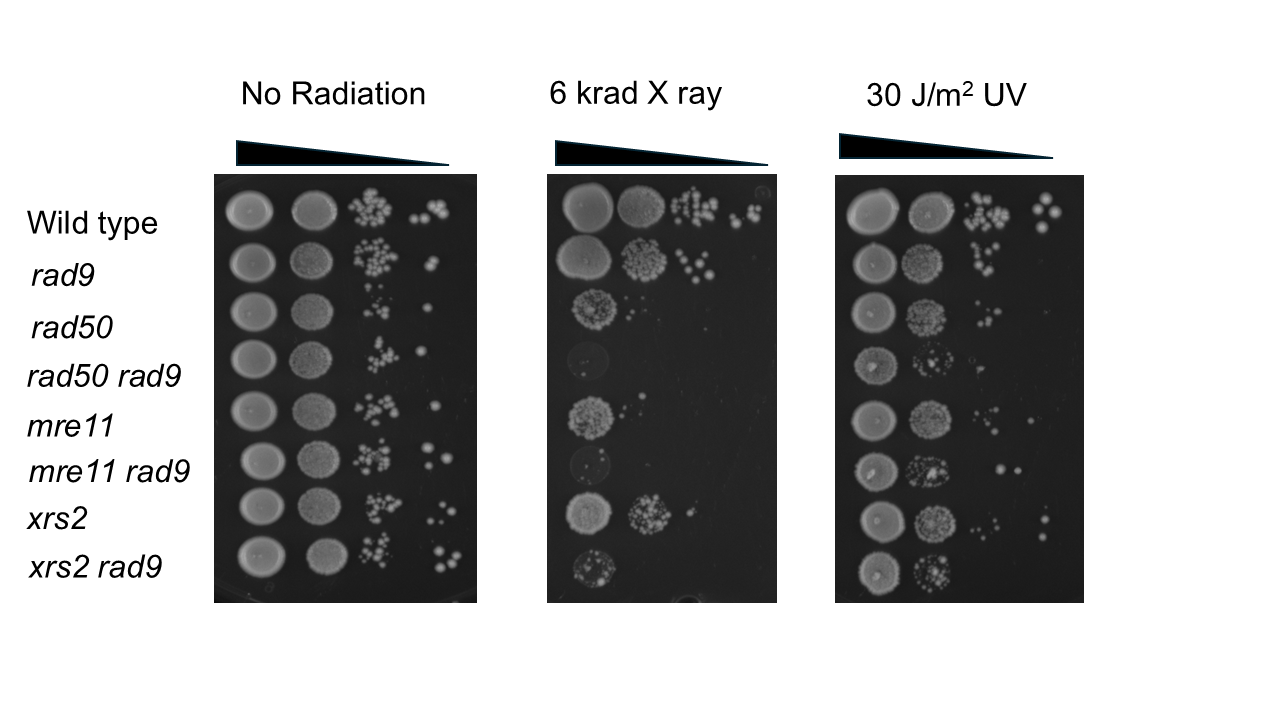

Supplement: Supplementary file 2 [file Image1.tif]
