# Supplementary material for: Checkpoint and recombination pathways independently suppress rates of spontaneous homology-directed chromosomal translocations in budding yeast
Source: Front Genet. 2025 Apr 4;16:1479307. doi: 10.3389/fgene.2025.1479307 (PMC12006765; doi:10.3389/fgene.2025.1479307)
Supplement: Supplementary file 3 [file Table2.docx]

Supplementary Table 2. Characterization of Colony Growth of Rad Diploid Cells 24 Hours After Irradiation (2.5 krads).

| Diploid Strain^1.^ | Single Unbudded Cells (%)^2.^ | Large-budded Cells | Microcolonies (2-100 cells) | Minicolonies(> 100cells) |
| --- | --- | --- | --- | --- |
| Rad^+^ | 6 (6) | 2 (2) | 15 (14) | 83 (84) |
| *rad55* | 4 (4) | 16 (15) | 47 (46) | 36 (35) |
| *mrell* | 16 (15) | 12 (12) | 61 (60) | 13 (13) |
| *xrs2* | 5 (5) | 3 (3) | 90 (88) | 4 (4) |
| *rad50* | 26 (21) | 6 (5) | 75 (61) | 15 (12) |
|  |  |  |  |  |
| *rad50 rad9* | 0 | 0 | 85 (82) | 19 (18) |
| *mre11 rad9* | 8 (7) | 3 (2) | 105 (88) | 4 (3) |
| *xrs2 rad9* | 2 (2) | 2 (2) | 81 (79) | 17 (17) |
| *rad51 rad9* | 1 (1) | 6 (6) | 87 (92) | 1 (1) |

^1.^ For full genotype, see supplemental Table 1.

^2.^ Percentage rounded to the nearest integer.
