# Supplementary material for: Checkpoint and recombination pathways independently suppress rates of spontaneous homology-directed chromosomal translocations in budding yeast
Source: Front Genet. 2025 Apr 4;16:1479307. doi: 10.3389/fgene.2025.1479307 (PMC12006765; doi:10.3389/fgene.2025.1479307)
Supplement: Supplementary file 4 [file Table3.docx]

SUPPLEMENTARY TABLE 3. Interaction Factors (IF) For Selected Double Mutants.

| Genotype (Strain) | G_1_G_2_ | G_1_ | G_2_ | C | IF^2.^ | Type of Interaction^3.^ |
| --- | --- | --- | --- | --- | --- | --- |
| *rad9 rad51* (YB749) | 176 | 17 | 30 | 4 | 133 | Synergistic |
| *rad9 rad55* (YB753) | 226 | 17 | 33 | 4 | 180 | Synergistic |
| *rad9 rad57* (YB754) | 199 | 17 | 34 | 4 | 152 | Synergistic |
| *rad9 rad54* (YB751) | 161 | 17 | 2.8 | 4 | 145.2 | Synergistic |
| *rad9 xrs2* (YB755) | 126 | 17 | 76 | 4 | 37 | Synergistic |
| *rad9 mre11* (YB756) | 166 | 17 | 81 | 4 | 72 | Synergistic |
| *rad9 rad50* (YB750) | 54 | 17 | 63 | 4 | -22 | Inhibition |
| *mec1-21 rad51* (YB757) | 288 | 30 | 65 | 3 | 196 | Synergistic |

^1.^ For full genotype, see Suplementary Table 1.

^2.^ IF = G _1_ G _2_ – G _1_ – G _2_ + C (David et al., 2016) , where G_1_G_2_ is the adjusted (net) recombination rate/10^8^ for the double mutant, G_1_ and G_2_ is the net recombination of the single mutants, and C is the recombination rate for the wild-type diploid strain. The net recombination rate/10^8^ was calculated by subtracting the rate observed in wild type from the rate observed in the mutant and multiplying by 10^8^.

^3.^ If IF is positive, then the interaction is synergistic and if negative the interaction is inhibition.
